# Irinotecan and Δ^9^-Tetrahydrocannabinol Interactions in Rat Liver: A Preliminary Evaluation Using Biochemical and Genotoxicity Markers

**DOI:** 10.3390/molecules23061332

**Published:** 2018-06-01

**Authors:** Ana Lucić Vrdoljak, Nino Fuchs, Anja Mikolić, Suzana Žunec, Irena Brčić Karačonji, Andreja Jurič, Ljerka Prester, Vedran Micek, Marijana Neuberg, Samir Čanović, Gordan Mršić, Nevenka Kopjar

**Affiliations:** 1 Institute for Medical Research and Occupational Health, HR-10001 Zagreb, Croatia; alucic@imi.hr (A.L.V.); akatic@imi.hr (A.M.); suzana@imi.hr (S.Ž.); ibrcic@imi.hr (I.B.K.); ajuric@imi.hr (A.J.); prester@imi.hr (L.P.); vmicek@imi.hr (V.M.); 2 University Hospital Centre Zagreb, HR-10000 Zagreb, Croatia; Ninofuchs84@yahoo.com; 3 University Centre Varaždin, University North, HR-42000 Varaždin, Croatia; marijana.neuberg@unin.hr; 4 Zadar General Hospital, HR-23000 Zadar, Croatia; canovicsamir@yahoo.com; 5 Forensic Science Centre “Ivan Vučetić”, HR-10000 Zagreb, Croatia; gmrsic@mup.hr

**Keywords:** cannabinoid-based preparations, functional liver impairments, genotoxicity, hepatocytes, liver to body weight ratio, oxidative stress

## Abstract

There is growing interest regarding the use of herbal preparations based on *Cannabis sativa* for medicinal purposes, despite the poorly understood interactions of their main constituent Δ^9^-tetrahydrocannabinol (THC) with conventional drugs, especially cytostatics. The objective of this pilot study was to prove whether the concomitant intake of THC impaired liver function in male Wistar rats treated with the anticancer drug irinotecan (IRI), and evaluate the toxic effects associated with this exposure. IRI was administered once intraperitoneally (at 100 mg/kg of the body weight (b.w.)), while THC was administered per os repeatedly for 1, 3, and 7 days (at 7 mg/kg b.w.). Functional liver impairments were studied using biochemical markers of liver function (aspartate aminotransferase—AST, alanine aminotransferase—ALP, alkaline phosphatase—AP, and bilirubin) in rats given a combined treatment, single IRI, single THC, and control groups. Using common oxidative stress biomarkers, along with measurement of primary DNA damage in hepatocytes, the degree of impairments caused at the cellular level was also evaluated. THC caused a time-dependent enhancement of acute toxicity in IRI-treated rats, which was confirmed by body and liver weight reduction. Although single THC affected ALP and AP levels more than single IRI, the levels of liver function markers measured after the administration of a combined treatment mostly did not significantly differ from control. Combined exposure led to increased oxidative stress responses in 3- and 7-day treatments, compared to single IRI. Single IRI caused the highest DNA damage at all timepoints. Continuous 7-day oral exposure to single THC caused an increased mean value of comet tail length compared to its shorter treatments. Concomitant intake of THC slightly affected the levels of IRI genotoxicity at all timepoints, but not in a consistent manner. Further studies are needed to prove our preliminary observations, clarify the underlying mechanisms behind IRI and THC interactions, and unambiguously confirm or reject the assumptions made herein.

## 1. Introduction

In recent years, we are witnesses to increased controversies associated with the use of *Cannabis sativa* products, which have slowly integrated into modern medicine. Despite insufficient knowledge on the therapeutic efficiency of such preparations, there is also rising concern about the possible undesirable adverse effects resulting from the pharmacokinetic and pharmacodynamics interactions of their main constituents with conventional medications, which still are poorly understood.

*Cannabis sativa* is characterized by a complex phytochemical profile, which comprises more than 400 chemicals [1]. Among them, the most active component is Δ^9^-tetrahydrocannabinol (THC) [2]. As a psychoactive compound [2], THC has a high abuse potential, so in most countries, the use of *C. sativa* products is still prohibited and illegal. Nevertheless, the multitude of effects they produce in an organism make *C. sativa* products attractive self-medications, used to relieve symptoms such as diarrhoea, abdominal pain, reduced appetite, chemotherapy-induced nausea, vomiting, etc. [3].

Many of the abovementioned symptoms commonly occur during chemotherapy based on the anticancer drug irinotecan (IRI). Irinotecan or 7-ethyl-10-[4-(1-piperidino)-1-piperidino] carbonyloxycamptothecin (also known as CPT-11) is one of the most important antineoplastic drugs developed in the last decades. It is a semisynthetic analogue of the plant alkaloid camptohecin [4,5]. Although primarily intended for the use in chemotherapy of metastatic colorectal cancer [6], IRI is also effective against lung cancer, ovarian cancer, leukaemia, malignant gliomas [4,7,8,9]. IRI is a pro-drug that has to be converted to the active metabolite SN-38 via carboxylesterases [10]. IRI and SN-38 belong to the class of DNA topoisomerase I inhibitors [6,11]. SN-38 is 100–1000 fold more cytotoxic, and possesses better antitumor potential than the parent compound [12]. Besides conversion into SN-38, parent IRI is metabolised in the liver. During phase I metabolism, mediated by the CYP3A4 enzyme, IRI is converted into 7-ethyl-10-[4-*N*-(5-aminopentanoic acid)-1-piperidino]carbonyloxycamptothecin (APC), and 7-ethyl-10-[4-(1-piperidino)-1-amino]-carbonyloxycamptothecin (NPC). Both compounds possess significantly lower cytotoxic potential [4,13,14]. SN-38 undergoes Phase II metabolism via glucuronidation, which is mediated by UDP-glucuronosyltransferase enzyme (UGT), isomer UGT1A1 [4]. A major route of IRI elimination is faecal excretion [4]. Due to enterohepatic recirculation, a part of glucuronidated SN-38 is converted back into SN-38 in the colon, by bacterial glucuronidases [15], and this phenomenon leads to delayed onset diarrhoea, which is a severe side-effect of the drug. Another potentially life threatening adverse effect is myelosuppression, which might lead to neutropenia [6,9,11]. Recently, more attention has been paid to IRI-mediated hepatotoxicity, as well [16,17,18,19].

Although there are various conventional therapies that can counteract or reduce the adverse effects of anticancer drugs, we are facing growing use of both legally prescribed preparations which contain cannabinoids (or medical marijuana), as well as illicit ones used for the same purpose. In contrast to approved and registered products, which contain a standardised and defined dose of THC, there is a huge problem with illicit ones that sometimes contain very high THC levels. This puts users under an increased risk of adverse effects, as well as of potential intoxication. Growing evidence indicates that cannabis preparations produced various effects in organism, some of which were not beneficial [20]. Badowski [3] recently reviewed that even approved oral cannabinoids are associated with greater incidence of adverse effects compared with conventional antiemetic therapy. Chronic THC exposure is definitely associated with hepatotoxicity and steatosis [21,22].

After oral administration, much of the THC is initially metabolised in the liver before it reaches the sites of action. It undergoes rapid metabolism via CYP2C9, CYP2C19, and CYP3A4 enzymes to the psychoactive metabolite 11-hydroxy-Δ^9^-THC (11-OH-THC), which is further oxidized to the inactive 11-nor-9-carboxy-Δ^9^-THC (THC-COOH) [23,24,25,26]. Phase II THC metabolites are mainly conjugates of phase I metabolites with glucuronic acid. This reaction is catalysed by UGTs. 11-OH-THC is a substrate preferentially metabolised by UGT1A9, but also recognised by UGT1A10. THC-COOH is a substrate recognised by UGT1A1 and UGT1A3 [24,25,27]. 

Although THC rapidly penetrates highly vascularised tissues, such as the liver, its major long-term storage site is body fat [25,28]. THC metabolites undergo extensive enterohepatic recirculation [25,29]. They are excreted within days and weeks, predominantly in faeces [25,28,30]. 

To date, several studies in rats have focused on the assessment of IRI hepatotoxicity, and especially, biliary excretion [18,31,32,33,34,35,36,37]. On the contrary, there is a limited number of studies that explored the potential hepatotoxicity of *Cannabis sativa* products using the rat model [38,39,40,41]. However, none of the studies conducted so far on rats investigated the effects of the concomitant use of IRI and single THC. This gap in knowledge motivated us to explore whether and how the concomitant intake of pure THC impaired liver function in IRI-treated rats, and evaluate the toxic effects associated with this exposure. To accomplish these goals, we established a simple experimental design on a rat model focused on biomarkers that point out acute toxicity and liver impairments at functional and cellular level. Functional liver impairments caused by treatments have been investigated using biochemical markers of liver function: aspartate aminotransferase (AST), alanine aminotransferase (ALT), alkaline phosphatase (ALP), and bilirubin. The degree of impairments caused by single compounds and their combination at cellular level was evaluated using common oxidative stress biomarkers, along with measurement of primary DNA damage in hepatocytes by the alkaline comet assay. 

To the best of our knowledge, no previous study focused on the same interactions on a rat model by employing a comparable study design and similar array of biomarkers. We hypothesised that the complex interplay between IRI and THC could lead to hepatotoxicity enhancement and had expected that a combination of selected biomarkers would give more insight into the potentially harmful interactions of the tested compounds as well as point to the directions in which future research should go. This pilot study focused solely on a rat model and took into account all of its limitations, as well as methodological constraints, without any intention to presume analogous outcomes in the humans.

## 2. Results and Discussion

**Figure 1:**
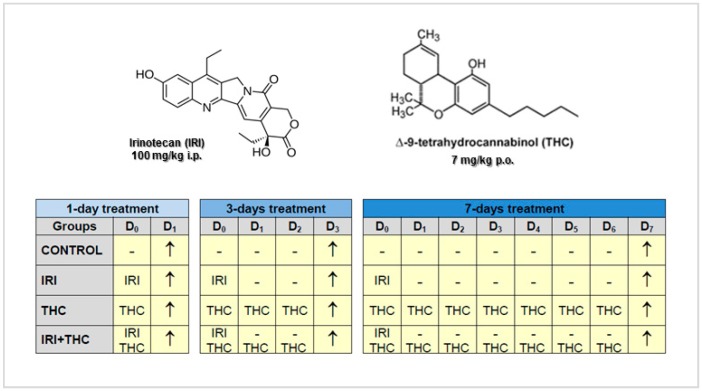
Structural formulas of irinotecan (IRI), Δ^9^-tetrahydrocannabinol (THC), and experimental schedule. Legend: i.p., intraperitoneal; p.o., per os; D, day; −, without treatment; ↑, termination of the experiment (sampling).

The experimental schedule included a single intraperitoneal application of IRI (at 100 mg/kg b.w.), while THC was administered per os repeatedly for 1, 3, and 7 days (at 7 mg/kg b.w.). The experiment was terminated 24 h after the last treatment, as shown in the timetable displayed in Figure 1.

None of the treatments resulted in death of the experimental animals. Such an outcome was anticipated, considering the tested dose, exposure route, and treatment duration we applied. The selected dose of 100 mg/kg b.w. of IRI per week was used in previous studies [42,43,44,45], and has been confirmed as the maximum tolerated dose for rats. It usually did not result with severe diarrhoea and gastrointestinal toxicity, and our observations were the same.

However, the range of IRI doses tested on rats so far, as well as the experimental schedules (including exposure duration and administration routes), is quite variable. Single daily doses of IRI tested in rats ranged from 2 mg/kg b.w. [46] to 300 mg/kg [47]. Most representative toxicity studies with IRI conducted on rats [47,48,49,50,51] evaluated the effects of a single dose of 200 mg/kg, which has low mortality, but caused reproducible gastrointestinal mucositis. 

The IRI doses used in humans are also quite variable, depending on the particular chemotherapy regimen [6,8,52]. Human doses are expressed based on the body surface area (which for the average person is 1.73 m^2^) [8], and not in mg/kg, as the doses used in the animal studies. Weekly IRI doses when the drug is given as monotherapy are 100–350 mg/m^2^, while in combination therapies, they are usually 80, 125, 165, and 180 mg/m^2^ [6,52]. However, the maximal and recommended tolerated human doses are 10–30 mg/m^2^/day [4].

From the abovementioned reports, it is clear that rats generally tolerated much higher daily doses of IRI than humans. Therefore, when we planned this study, to make sure that the tested IRI dose would produce a magnitude of effects measurable with the applied methods, we intentionally selected a dose whose toxic effects were established in previous studies on rats. In spite of the fact that the tested dose was higher than the doses used in humans, we believe that this study design was appropriate, since we focused only on a rat model, which enabled us to control for confounding factors. We are highly aware that the findings obtained in one narrow pilot study on rats could not directly and simply be extrapolated to real life situation involving humans, especially having in mind the complexity of all of the possible interspecies differences in pharmacokinetics and pharmacodynamics of both of the tested compounds.

The choice of exposure route we applied was also based on existing rodent studies. The i.p. exposure route was used since this type of IRI administration has been shown to be more effective and less toxic to rodents [4,53] than the intravenous route, which is usually used in humans. As known, i.p. administration leads to the absorption of the tested compound through the portal circulation, which must pass through the liver before reaching systemic circulation and other organs [54,55]. This is, in the case of IRI, particularly important considering that it is a pro-drug that has to be converted to the active metabolite SN-38. 

We have to stress that IRI is rarely delivered via the oral route, mostly due to its poor and variable bioavailability and high gastrointestinal toxicity [4,35]. Several studies on rats evaluated IRI bioavailability after oral exposure, and found that it could be improved through the use of biliary excretion inhibitors, such as verapamil and quercetin [35,56,57], which also led to decreased toxicity. There have also been attempts to design a new delivery systems for IRI (liposomes, micelle solutions, microemulsions, etc.) [58], as well as to design nanoparticle drug formulations [59], which could ensure high systemic concentrations of the drug without producing severe gastrointestinal toxicity. 

The fact that no related studies on rat model have been conducted with “pure” THC so far, made the choice of the representative dose difficult. The dose of 7 mg/kg b.w. we tested was selected based on the available literature reports regarding various illicit preparations rich in THC [60,61,62]. In contrast to our study, most existing studies on rat model [38,40,41,63,64] evaluated the effects of *Cannabis sativa* extracts, rather than THC. This, to a certain degree, complicates the interpretation of results, since *Cannabis sativa* contains over 400 bioactive compounds [1]. Since THC has a somewhat higher bioavailability if consumed in an oil formulation [65], we applied the same formulation and selected the oral route of THC exposure. Another reason for this was the fact that approved medications containing synthetic THC analogues, like dronabinol, are also given per os [3,66,67]. Similarly as in the case of IRI, much of the THC is initially metabolised in the liver before it reaches the sites of action [25].

Considering the specificity and sensitivity of the biomarkers used in this study, we selected three sampling times: one, three, and seven days after the administration of IRI. After one day, we expected to detect early signs of toxicity, especially at the level of oxidative stress biomarkers and DNA. Taking into account known facts on IRI-associated mechanisms of action at cell level, three days after IRI application, we expected to detect potentially hepatotoxic effects using all endpoints. On the other hand, at this timepoint, possible toxic effects of single THC, as well the outcomes of its interactions with IRI (or their metabolites’ interactions) could be also detectable. Finally, the 7-day timepoint was selected, keeping in mind that repeated exposure to THC could result in the formation of many metabolites which might interfere with all processes at cell level, and influence the functional integrity of the rat liver as well.

**Figure 2:**
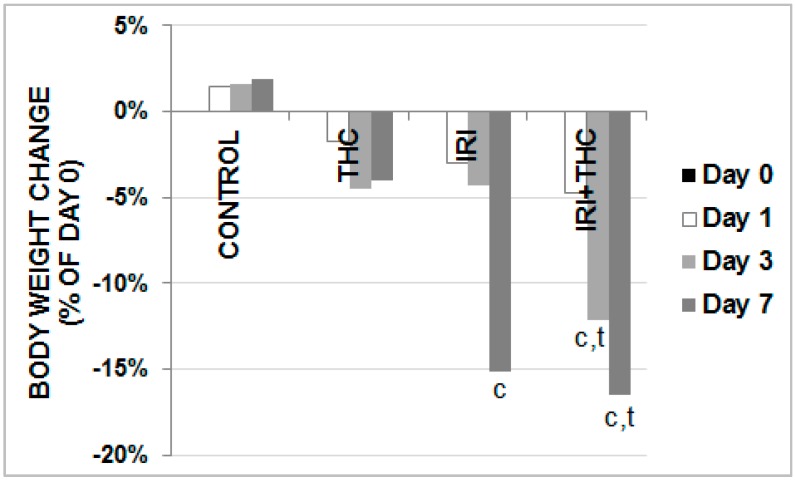
Changes in body weight gains in male Wistar rats after the 1-, 3-, and 7-day treatments with IRI, THC, their combination (IRI + THC), and in the respective controls. Values are expressed as mean ± SD (*N* = 5). Significantly different (*p* < 0.05) values were c: vs. control; t: vs. THC.

We first focused on the observed systemic effects of the tested compounds. At the end of each treatment, the body weight of rats was measured and compared with the initial body weight value. Results regarding changes in body weight gains are reported in Figure 2.

In all of the treated rats, a time-dependent reduction in body weight gain was observed. Following IRI administration, body weight started to significantly decrease after three days. The most prominent body weight reduction was observed in rats administered a combined treatment. As shown in Figure 2, it was statistically significant compared to respective controls both after 3- and 7-day long treatments, while at the end of the experiment, these rats also had lower body weight gain compared to the THC-exposed ones.

Considering that the rats had free access to food and water during the experiment, treatment-related distress possibly reduced the rats’ appetite. Furthermore, the weight loss in IRI-exposed rats could also be related to its detrimental effect on intestinal absorption. As known, enterohepatic recirculation leads to the return of glucuronidated IRI metabolite SN-38G s into active form, via bacterial β-glucuronidase activity. Due to deconjugation, levels of highly cytotoxic SN-38 increased and directly damaged the intestinal epithelium, which was confirmed in previous studies [46,68,69,70]. 

**Figure 3:**
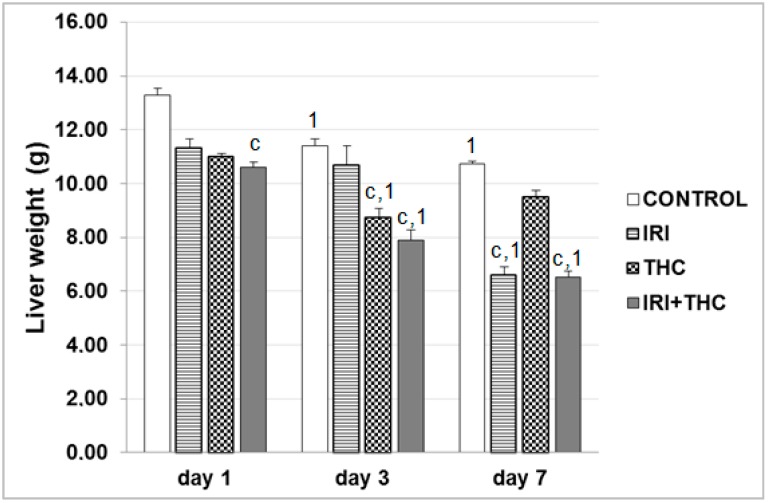
Liver weight changes in male Wistar rats after the 1-, 3-, and 7-day treatments with IRI, THC, their combination (IRI + THC), and in the respective controls. Values are expressed as mean ± SD (*n* = 5). Significantly different (*p* < 0.05; ANOVA, post hoc Tukey HSD test) values were c: vs. control; t: vs. THC; 1: vs. 1-day treatment.

Results regarding liver weight changes are reported in Figure 3. Combined treatment had the most detrimental effects on liver weights, indicating a potentiation of IRI hepatotoxicity by THC, and caused their statistically significant decreases compared to control at all of the timepoints. Other significant differences are shown in Figure 3.

**Figure 4:**
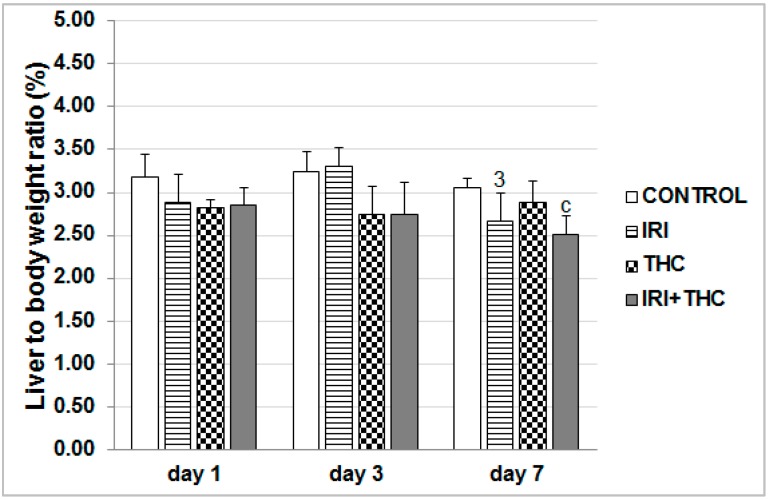
Liver to body weight ratio in male Wistar rats after the 1-, 3-, and 7-day treatments with IRI, THC, their combination (IRI + THC), and in the respective controls. Values are expressed as mean ± SD (*n* = 5). Significantly different (*p* < 0.05; ANOVA, post hoc Tukey HSD test) values were c: vs. control; 3: vs. 3-day treatment.

Results regarding liver to body weight ratios are displayed in Figure 4. The lowered body weights in the treated rats mostly resulted in a similar trend of lowered liver weights. This is well established in the literature [71]. At all of the timepoints, the combined treatment mostly lowered liver to body weight ratio, which was statistically significant compared to control only after 7-day treatment. IRI effects on liver weight reduction could be partly explained by the loss of hepatocytes, due to drug cytotoxicity. This is supported by the finding of increased levels of aminotransferases measured in serum, which are sometimes associated with enzyme leakage from damaged hepatocytes [72,73]. Furthermore, the apoptotic potential of the tested drug also has to be taken into account. Due to its main mechanism of action, IRI leads to the formation of double-strand DNA breaks that are generally non-repairable, and which lead to apoptosis [74,75]. In addition, the role of apoptosis in liver impairments has also been well-documented previously [76].

Taken together, results regarding body weight and liver weight changes observed after treatments suggest that both single IRI and THC, and their combination at the tested doses and applied experimental schedule produced acute toxicity, which led to changes in the overall fitness of the exposed vs. control rats. To determine whether the observed toxic effects were related to functional liver deficiencies, or to the impairments of different essential processes at cellular level, we performed detailed analyses in that regard.

### 2.1. Serum Parameters of Liver Function

#### 2.1.1. AST, ALT, and ALP Activity

To establish the level of functional liver impairment after exposure to tested compounds, we studied changes in the activities of AST, ALT, and ALP. Increased serum activities of ALT and AST generally point to the liver tissue injury or cell damage which resulted in the leakage of the enzymes into the extracellular matrix and circulation [72,77]. Although serum ALP increases in liver injuries, its values may also increase in bone disorders, heart failure, and bacterial infection [72,78].

**Figure 5:**
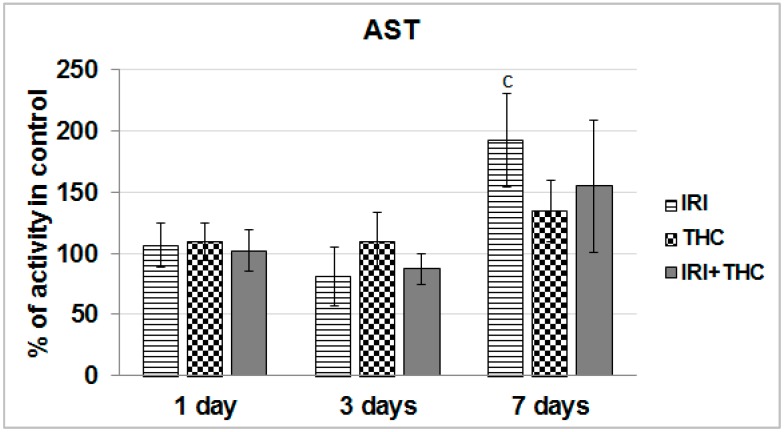
Effects of 1-, 3-, and 7-day treatments with irinotecan (IRI), Δ^9^-tetrahydrocannabinol (THC), and their combination (IRI + THC) on aspartate aminotransferase (AST) activity in the serum of male Wistar rats. Values are expressed as mean ± SD (*n* = 5). The significantly different (*p* < 0.05) value was c: vs. control rats.

We found that AST activity was significantly increased compared to respective control only in IRI-treated rats after the 7-day treatment (Figure 5). 

**Figure 6:**
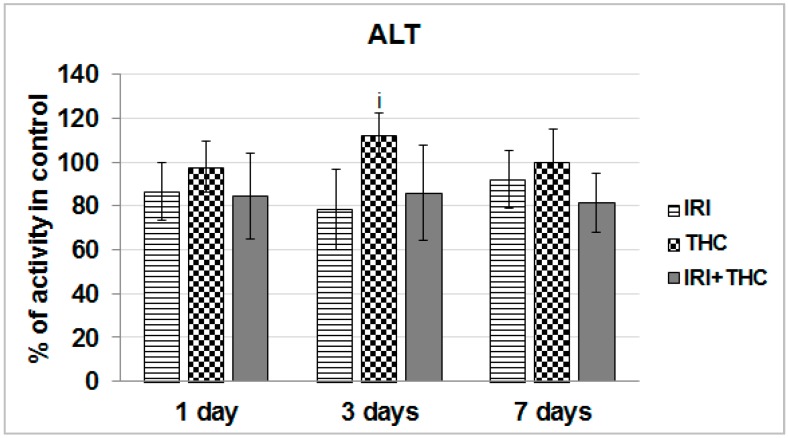
Effects of 1-, 3-, and 7-day treatments with irinotecan (IRI), Δ^9^-tetrahydrocannabinol (THC), and their combination (IRI + THC) on alanine aminotransferase (ALT) activity in the serum of male Wistar rats. Values are expressed as mean ± SD (*n* = 5). The significantly different (*p* < 0.05) value was i: vs. IRI-treated rats.

As shown in Figure 6, a statistically significant increase of ALT activity with respect to IRI-treated rats was observed only in THC-treated rats after the 3-day exposure.

**Figure 7:**
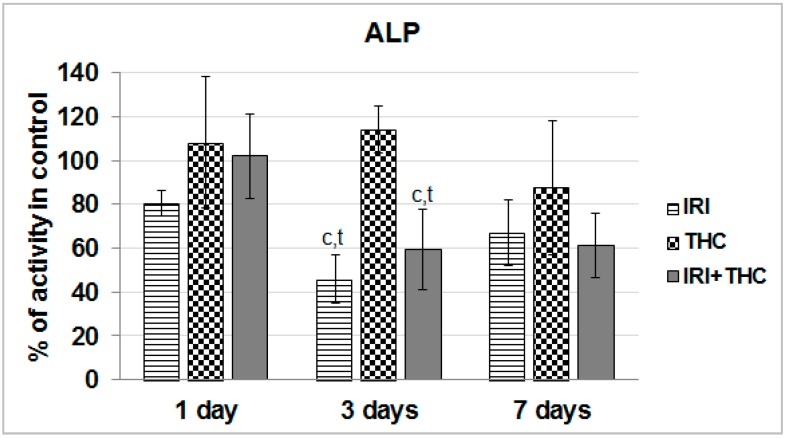
Effects of 1-, 3-, and 7-day treatments with irinotecan (IRI), Δ^9^-tetrahydrocannabinol (THC), their combination (IRI + THC) on alkaline phosphatase (ALP) activity in the serum of male Wistar rats. Values are expressed as mean ± SD (*n* = 5). The significantly different (*p* < 0.05) value was c: vs. control rats.

Exposure to single IRI caused a statistically significant decrease of serum ALP activity compared to control and THC-treated rats after the 3-day treatment (Figure 7). 

Taken together, the obtained results suggest that liver function was relatively well-preserved after only one application of IRI. As known, the liver has a great capacity to heal damage, and a sort of adaptive tolerance is also possible [79]. What calls for some concern is the increased AST level measured in IRI-treated rats after the 7-day treatment. However, since this enzyme is located in other tissues as well, including kidney, heart, brain, and skeletal muscle [72,80], increased AST could not be linked exclusively to liver damage. 

We also have to stress that after repeated 7-day oral exposure of Wistar rats to single THC, a trend towards increased AST activity was observed at all three of the timepoints, and ALT level was somewhat increased at day 3, and ALP level was increased at days 1 and 3. It is possible that levels of serum biomarkers temporarily increased, as they reflect both the rate of their release from hepatocytes and the rate of plasma clearance. Nevertheless, since this pilot study tested only one THC dose, further studies are necessary. 

Combined exposure resulted in relatively small functional impairments of serum liver markers. However, it is hard to accurately estimate functional liver impairments after testing the effects of only one combination of doses, and this result has to be taken with precaution. The subject is definitely worth further investigation, since there are indications that THC-rich *C. sativa* extract administered concomitantly to rats with two hepatotoxins, acetaminophen and CCl_4_, enhanced acute hepatic damage [38].

Not many rodent studies up to now evaluated the same biomarkers of liver function after exposure to IRI, but they reported generally similar results [36,81,82,83]. The existing literature on serum markers of rodent hepatocellular damage after exposure to THC is inconsistent [38,39,40,41,63,64,84]. It is pretty hard to compare and justify our results with previously published studies, mostly due to different routes of exposure, treatment duration, but also the use of *C. sativa* extract instead of pure THC, which was tested here. However, most of the above mentioned studies did not indicate significant impairments of AST, ALT, and ALP levels after an intake of *C. sativa* preparations.

#### 2.1.2. Total Protein in Serum

**Figure 8:**
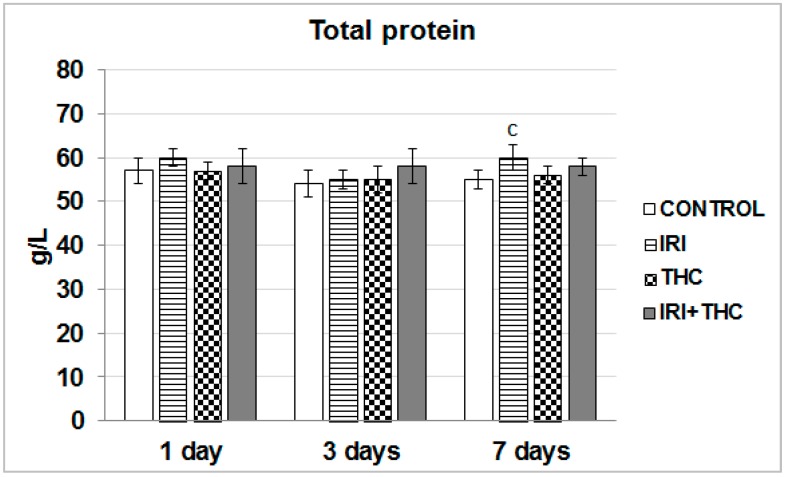
Effects of 1-, 3-, and 7-day treatments with irinotecan (IRI), Δ^9^-tetrahydrocannabinol (THC), and their combination (IRI + THC) on total protein in the serum of male Wistar rats. Values are expressed as mean ± SD (*n* = 5). The significantly different (*p* < 0.05) value was c: vs. control rats.

Serum total protein level is recognized as an indicator of the functional status of liver cells [85]. As shown in Figure 8, a statistically significant increase in the amount of total protein compared to the respective control was found only in the IRI group after the 7-day treatment. 

It has been well established that both IRI and THC metabolites bind to plasma proteins. SN-38 mainly binds to albumin [4]. THC is mainly bound to lipoproteins and less to albumin [25,86]. Since the largest part of total protein refers to the concentrations of albumin and immunoglobulins, without conducting specific tests, it is hard to conclude which of them contributed more to the overall increase. Nevertheless, as the liver has large reserves of albumin synthetic capacity [80], we found it rather unlikely that a single application of IRI could lead to a significant impairment in albumin amounts. It is most likely that the IRI treatment activated the host inflammatory responses to a greater extent than it affected small molecule transport. However, this has to be clarified in future studies.

#### 2.1.3. Bilirubin

Measurement of bilirubin levels is a valuable diagnostic marker of liver disorders [78]. Existing data offer very limited information on bilirubin levels measured after exposure to single IRI and single THC, while their combined effects have not been studied so far. Only one study, by Gupta et al. [87] measured the level of plasma total bilirubin in rats pre-treated with 60 mg/kg of cyclosporine A before i.v. administration of IRI at 6 mg/kg. They did not find a significant increase in total bilirubin between control and pre-treated rats. In a study on Wistar rats treated orally with *C. sativa* extract at 1 and 3 mg/kg b.w. for 30 days, Okwary et al. [41] found significantly increased total bilirubin levels compared to controls.

**Figure 9:**
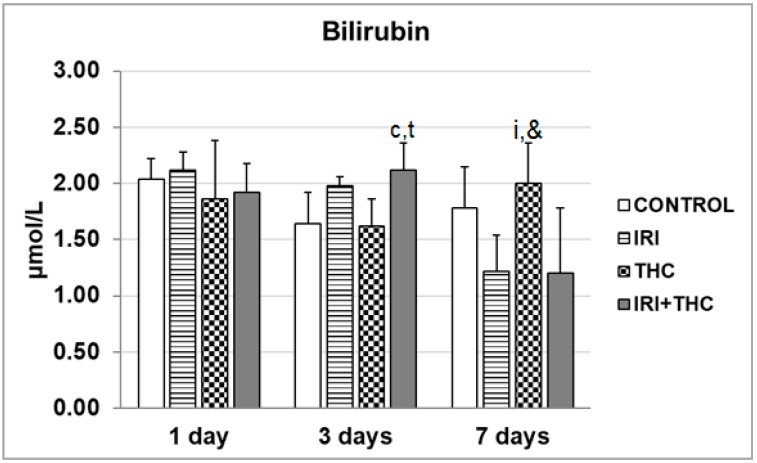
Serum bilirubin levels measured in male Wistar rats after the 1-, 3-, and 7-day treatments with irinotecan (IRI), Δ^9^-tetrahydrocannabinol (THC), their combination (IRI + THC), and in the respective controls. Values are expressed as mean ± SD (*n* = 5). The significantly different (*p* < 0.05) values were c: vs. control; i: vs. IRI-treated rats; t: vs. THC-treated rats; &: vs. rats treated with IRI and THC.

Results regarding serum bilirubin levels measured in the present study are shown in Figure 9. The combined treatment resulted in a statistically significant increase of serum bilirubin level only after the 3-day treatment, compared to the respective control and THC groups. It is worth mentioning that seven days of continuous intake of single THC resulted with a serum bilirubin level higher than its levels observed in rats administered single IRI, and a combined treatment (Figure 9). The significance of this particular finding has to be investigated in more detail in future studies. Since it is well known that the same UGT1A1 enzyme isoform is responsible for glucuronidation of bilirubin, SN-38, and THC-COOH [5,14,25,27,88,89,90,91], their mutual interactions also have to be clarified using more subtle biomarkers, which we, unfortunately, were not able to demonstrate due to methodological constraints.

### 2.2. Biochemical Markers of Oxidative Stress

The present study evaluated the extent of lipid peroxidation by measuring the level of thiobarbituric acid reactive substances (TBARS). As known, TBARS assay represents one of the most widely used assays for the determination of malondialdehyde, a reactive lipid peroxidation end product [92]. We also measured the activities of intrinsic antioxidant enzymes superoxide dismutase (SOD) and catalase (CAT). SOD catalyses the dismutation of the highly reactive superoxide anion to O_2_, and to the less reactive species hydrogen peroxide [93], while CAT prevents cell oxidative damage by converting hydrogen peroxide into water and oxygen [94].

**Figure 10:**
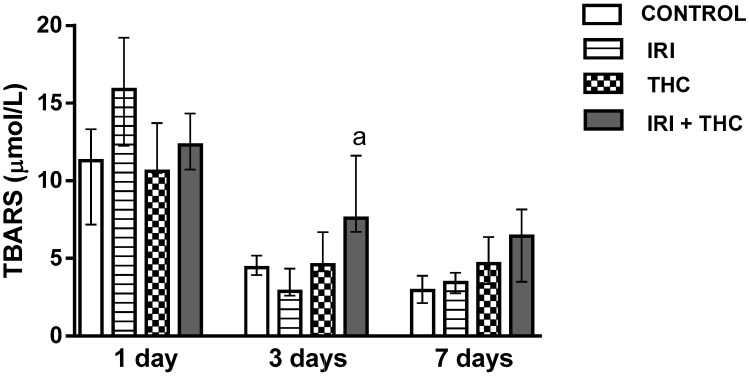
Changes in the thiobarbituric reactive substances (TBARS) concentration in the liver of rats after the 1-, 3-, and 7-day treatments with irinotecan (IRI), Δ^9^-tetrahydrocannabinol (THC), their combination (IRI + THC), and in the respective controls. The results are shown as median and interquartile range. The significantly increased values (*p* < 0.05) were (a) compared to rats treated with IRI.

**Figure 11:**
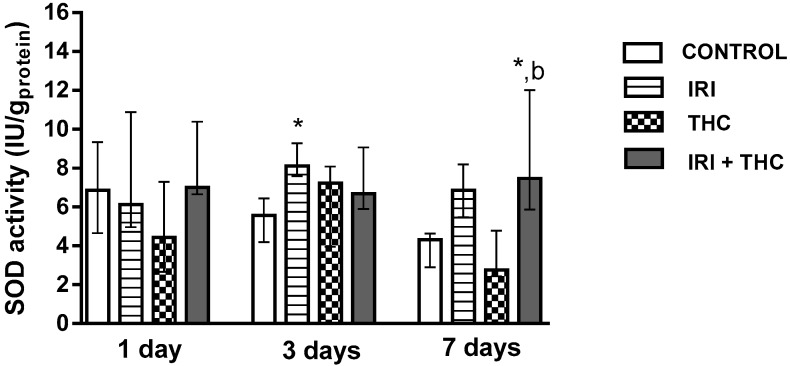
Changes in superoxide dismutase (SOD) activity in the liver of rats after the 1-, 3-, and 7-day treatments with irinotecan (IRI), Δ^9^-tetrahydrocannabinol (THC), their combination (IRI + THC), and in the respective controls. The results are shown as median and interquartile range. The significantly increased values (*p* < 0.05) were: (*) compared to control, (b) compared to rats treated with THC.

**Figure 12:**
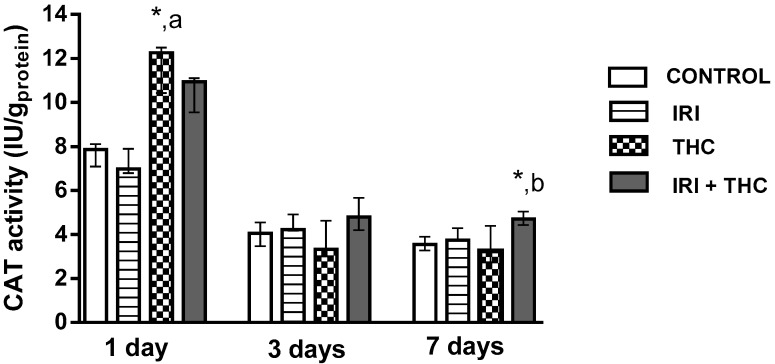
Changes in catalase (CAT) activity in the liver of rats after the 1-, 3-, and 7-day treatments with irinotecan (IRI), Δ^9^-tetrahydrocannabinol (THC), their combination (IRI + THC), and in the respective controls. The results are shown as median and interquartile range. The significantly increased values (*p* < 0.05) were (*) compared to control, (a) compared to rats treated with IRI, (b) compared to rats treated with THC.

Results regarding levels of oxidative stress biomarkers measured in hepatocytes are reported in Figure 10, Figure 11 and Figure 12. Our findings, in general, showed that oxidative stress biomarkers sensitively detected toxic effects of all of the tested compounds, both when administered as single compounds or in combination.

#### 2.2.1. Lipid Peroxidation

Figure 10 shows changes in the concentration of TBARS measured in the liver of treated rats. It is notable that 3- and 7-day long treatments resulted in lower TBARS concentrations in all of the groups when compared to 1-day treatments. One day following application, single IRI produced the highest level of lipid peroxidation, while at the other two timepoints, the differences between IRI-treated and control rats were not significant (Figure 10). After the 3-day treatment, combined treatment resulted in statistically higher TBARS concentration compared to treatment with IRI alone.

Existing reports suggest increases of IRI-potentiated lipid peroxidation in the liver of CDF1 mice administered IRI i.p. at 100 mg/kg for six days [95]. Coşkun and Bolkent [39] found that i.p. exposure of Sprague-Dawley rats to 3 mg/kg b.w. THC for seven days resulted in slightly elevated lipid peroxidation in the liver.

#### 2.2.2. Superoxide Dismutase (SOD)

Figure 11 shows changes in the SOD activity in the liver of treated rats. After 3 days, SOD activity was significantly higher in the liver of rats treated with IRI compared to control. This possibly occurred as a response to the disturbed oxidative/antioxidative equilibrium due to treatment. Co-administration of THC with IRI resulted in a significant increase of SOD activity only after the 7-day exposure, compared to control and THC-treated rats. 

The available literature regarding SOD activity after IRI and THC exposure is scarce. Since there are no similar studies which evaluated SOD activity in the liver after IRI treatment, we cannot justify our findings. An earlier study with THC [39] found that i.p. exposure of Sprague-Dawley rats to 3 mg/kg b.w. THC for seven days resulted in a non-significant decrease in liver SOD activity compared to control rats.

#### 2.2.3. Catalase (CAT)

Figure 12 shows changes in the CAT activity in the liver of treated rats. One day after application, a single THC dose significantly increased CAT activity compared to control and IRI-treated rats. Combined treatment caused a significant increase of CAT activity compared to control and THC-treated rats. Such results may indicate the production of higher amounts of hydrogen peroxide, which then stimulated the enhanced enzyme activity. There were no related studies on IRI-treated rats where liver CAT activity was evaluated. Only one study [39] reported that i.p. exposure of Sprague-Dawley rats to 3 mg/kg b.w. THC for seven days resulted in decreased liver CAT activity compared to control rats.

With regard to the mechanisms responsible for oxidative stress after exposure of rats to IRI and THC, available literature suggests that they are associated with the effect on mitochondrial respiratory chain functions. Some reports suggest that IRI application might lead to mitochondrial dysfunction [16,19,96]. A disturbed expression of critical proteins important for electron transport encoded by mitochondrial DNA (mtDNA) leads to a damaged mitochondrial respiratory chain. This ultimately results in a vicious cycle of reactive oxygen species (ROS) generation, and might be the reason behind increased oxidative stress [97]. THC is also capable of inducing disruption of mitochondrial function and cell energetics, leading to increased oxidative stress [98,99]. It is worth mentioning that toxicity to hepatocyte mitochondria is also associated with the development of fatty liver (steatosis), which has been well established for both IRI [17,18,36,83] and THC [21,100].

### 2.3. Primary DNA Damage in Rat Hepatocytes

The DNA damaging potential of IRI in vivo has been established well, in contrast to THC. No similar studies with THC on a rat model are available in order for us to compare and justify our results. Due to the inhibition of enzyme DNA topoisomerase I, the main mechanism responsible for IRI-induced genotoxicity [4,6,9,11], exposure to this drug, generally results in excessive DNA damage.

Detection and quantification of primary DNA damage could be easily accomplished using the alkaline comet assay. In the last three decades, this method has been widely used for the assessment of primary DNA damage, primarily due to its versatility, sensitivity, and ability to detect the broad spectrum of DNA damage, ranging from DNA breaks, alkali-labile sites, DNA–DNA and DNA–protein crosslinks, etc. [101,102,103,104]. Its usefulness in detecting DNA damage following exposure to IRI was also confirmed in several studies performed on IRI-exposed rodents. Most previous studies were focused on measuring IRI-induced DNA damage in leukocytes [105,106], bone marrow [107], or colon cells [44,45]. In our previous study [108], we detected a significant increase in primary DNA damage in liver cells both at 24 h and 72 h following treatment of rats with an eleven-fold lower IRI dose than tested here.

**Figure 13:**
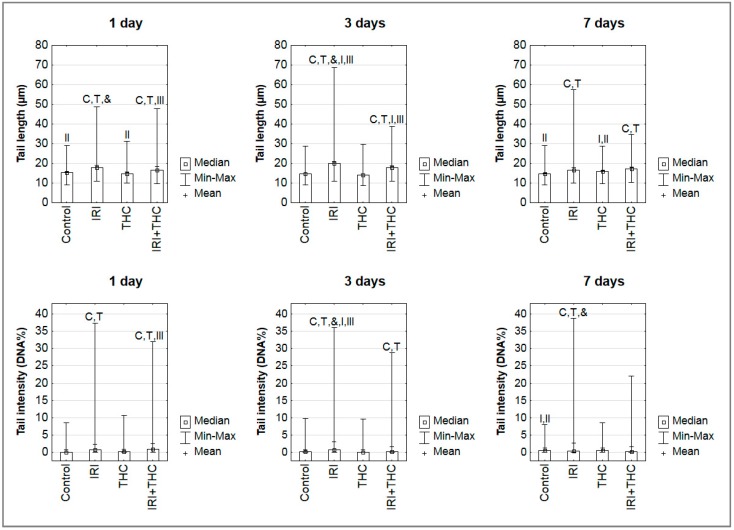
The levels of primary DNA damage measured in hepatocytes of male Wistar rats using the alkaline comet assay after 1-, 3-, and 7-day treatments with irinotecan (IRI), Δ^9^-tetrahydrocannabinol (THC), their combination (IRI + THC), and in the respective controls. Legend: IRI was administered once, via an intraperitoneal injection at a dose of 100 mg/kg b.w.; THC was dissolved in sesame oil and administered daily per os at a dose of 7 mg/kg b.w.; control rats were given the same daily volume of sesame oil as the THC group. Data are reported as mean/median values, and ranges (Min.—minimum value; Max.—maximum value) of one thousand independent comet measurements per each experimental group (200 comets/rat were scored on duplicate slides). Significantly different (*p* < 0.05, ANOVA with post hoc Tukey HSD test) compared to C—control; T—THC; &—combination of IRI and THC; I—1-day treatment; II—3-day treatment; III—7-day treatment.

Results of the alkaline comet assay obtained in the present study are reported in Figure 13.

Our observations and results obtained on IRI-treated rats are generally comparable with the existing studies mentioned above. After 1-day treatment, single IRI was more efficient in producing primary DNA damage compared to single THC, and the combined treatment. At this timepoint, a single dose of 100 mg/kg b.w. IRI induced significantly increased mean values of both tail length and tail intensity in rat hepatocytes, compared to the control and THC groups. Combined treatment induced significantly higher levels of primary DNA damage in hepatocytes compared to control and THC groups. After 3-day treatment, the group that received single IRI again had significantly increased mean values of both tail length and tail intensity compared to the control and THC groups. Rats administered combined treatment had significantly lower levels of primary DNA damage in hepatocytes compared to those given IRI, however, the measured values were still significantly higher than in the control or THC groups. After 7-day treatment, the highest mean value of tail intensity was measured in rats administered single IRI, followed by rats administered a combination of IRI and THC. More details regarding intergroup differences are shown in Figure 13.

Time-dependent changes in the DNA damage detected in the hepatocytes of single IRI-treated rats are consistent with the mechanisms that account for IRI toxicity. Since the parent drug has a terminal half-life of about 13 h, and a lower cytotoxic potential than its metabolite SN-38 [7], it is not surprising that the level of primary DNA damage measured using the alkaline comet assay after 1-day treatment was lower than after 3 days. Nevertheless, both the parent drug and SN-38 induced either directly or indirectly (via potentiating oxidative stress) many primary DNA lesions that could be easily detected by the alkaline comet assay even after a short exposure, which points to the high sensitivity of the method applied.

With the prolonged treatment duration, the level of DNA damage slightly diminished. This is certainly due to the fact that IRI was given once, and after seven days, the direct effects of the parent compound are no longer expected. A large part of DNA damage measured at this day could be related to indirect effects mediated via free radicals, as suggested by the measured levels of the oxidative stress markers, rather than IRI or SN-38. It also has to be stressed that after seven days equilibrium between DNA damage infliction and repair might occur, which contributed to the values measured by the comet assay.

Since there are no comet assay studies on THC-treated rats, we cannot compare and accurately justify our findings. We performed an extensive literature search regarding THC mutagenicity and genotoxicity, and found limited data. Genotoxicity of THC, as a single compound, has been poorly investigated using classical mutagenicity/genotoxicity assays. More reports refer to the genotoxicity of marijuana/cannabis. A few review articles [109,110,111] provide data on existing studies with cannabis and cannabinoids, whose results are rather inconsistent. In a recently published review, Reece and Hulse [112] stated that low doses of THC (<5 µg/mL or <5 µmol/L, or <1 joint/day) are usually not associated with genotoxic adverse outcomes. As it stands, we were first to use the comet assay aimed at evaluating genotoxicity of single THC on rat model. The available literature offers only one in vitro study [113], which used the same method to estimate the genotoxicity of marijuana smoke condensates in lung cancer cells.

What could be concluded from the results obtained here is that shorter exposures of rats to THC were not associated with a significant risk for genotoxicity, while continuous 7-day oral exposure caused minor DNA impairments in hepatocytes (significantly higher mean value of tail length, Figure 13). A possible reason why we detected such a pattern of DNA damage in the THC-treated rats was that, during the 7 days of study, the rats repeatedly received THC whose overall turnover and clearance of metabolites from the organism is slow, as known from previous pharmacokinetics studies [25,30,114]. The treatment schedule we applied caused a constant delivery of new amounts of the tested compound. Rapid metabolism of THC results in the formation of many metabolites (with an unknown inherent potency to induce primary DNA damage), which also have a different half-life in the body [29,30,88,114]. Upon their metabolism, other potentially DNA reactive products are formed also, which was confirmed using oxidative stress biomarkers. Furthermore, since THC is highly lipophilic [25,115], repeated 7-day exposure of rats leads to its accumulation in body fat. Its slow release from fat stores is also described as a source of potential “reintoxication” [115,116] that also generates potentially DNA-reactive compounds. As our study was limited with the selection of only one THC dose, the potentially detrimental effects of THC at DNA level have to be studied in more detail on a broader dose range. 

Results regarding comet tail intensity measured after 1-day treatment in liver cells of rats given a combined treatment (Figure 13) suggest that THC co-administration slightly enhanced IRI genotoxicity, which might have contributed to cytotoxic effects as well. It is interesting to note that, at all three timepoints, the maximum values measured for each comet parameter in rats administered a combined treatment were somewhat lower than the maximum values of tail lengths and intensities measured in rats given single IRI. This is an interesting finding, which could be associated with the presence of apoptotic cells. As known, the comet assay is based on the evaluation of DNA damage in single cells. Scoring criteria propose avoiding the measurement of nucleoids outside the measurement capabilities of the image analysis system [117]. Since apoptotic cells possess highly fragmented nucleoids, the conditions of the comet assay, including alkaline lysis, denaturation, and electrophoresis each can facilitate a “wash out” of their DNA from the agarose gel. Cells at later stages of apoptosis could especially be lost from comet scoring if they are not scored visually and recorded alongside the results of image analysis. In such cases, the measured values of tail length and intensity are based only on the remaining “more resistant” cells, and the obtained values could be lower than the actual damage. The influence of apoptosis on overall DNA damage levels has previously been established [118,119]. Therefore, we cannot exclude the possibility that the overall damage level in the hepatocytes of rats receiving a combined treatment was even higher than measured, due to the presence of apoptotic cells that “escaped” measurement.

Our study has some limitations as well. First of all, since this was an introductory study, we tested only one IRI and THC dose. As mentioned before, the tested IRI dose was higher than the doses used in humans, since it was selected based on previous rodent studies, by taking into account the sensitivity of the methods and biomarkers we used. Furthermore, our exposure schedule enables to evaluate only the early detrimental effects of the treatments. However, in a real-life situation, IRI chemotherapy is administered in regimens that are constituted of more than one cycle, and last for several weeks. Our results, therefore, are valid only within the frame of the proposed model, and were not intended to provide any clinically useful information at this preliminary stage of study.

We are aware that there are several questions that remain open, since the methods used in this study could not give definite answers about the mechanisms behind IRI and THC interactions; they could only suggest whether there were potentially interesting interactions that could be further clarified. Bearing in mind that this was an introductory study, and our plan is to extend the investigation within this research field, in the forthcoming studies, we would certainly find a way to overcome most of the limitations of the present experiment, and collect results that would unambiguously confirm or reject the assumptions made in this pilot study.

It should also be stressed that results obtained on rat model, although valuable and informative, cannot be directly extrapolated to real cancer patient scenarios. Before making any conclusion relevant for human risk assessment, one has to take into account the metabolic differences between the rat and human metabolism. Nevertheless, the results regarding IRI genotoxicity can be considered rather accurate, since the mechanisms of DNA damage induced by this compound both in rats and humans are the same.

## 3. Materials and Methods

### 3.1. Chemicals and Reagents

Irinotecan (CAS-No. 100286-90-6, provided as the hydrochloride trihydrate salt) was purchased from LC Laboratories (Woburn, MA, USA). Δ^9^-tetrahydrocannabinol (CAS-No. 1972-08-3) was purchased from THC Pharm GmbH (Frankfurt, Germany).

If not specified, other chemicals and reagents used in the experiment were purchased from Sigma-Aldrich Laborchemikalien GmbH (Steinheim, Germany).

### 3.2. Animals

Adult 3-month-old male Wistar rats were obtained from the breeding colony at the Institute for Medical Research and Occupational Health, Zagreb (Croatia). Animals were maintained under pathogen-free conditions in steady-state micro environmental conditions, 12 h light/dark cycle, 22 °C with ad libitum access to standard Good Laboratory Practice (GLP) certified food (Mucedola, 4RF21, Italy) and tap water. Appropriate enrichment was provided in animal cages. Use of animals must have been approved by the Institution’s Animal Care and Use Committee (IACUC). Protocols for the care, treatment, and euthanasia of the rats were approved by the Ethics Committee of the Institute for Medical Research and Occupational Health, Zagreb, Croatia (approval number: 100-21/16-16, 30 June 2016). The experiment was carried out in compliance with the principles expressed in the National Institutes of Health, USPHS, Guide for the Care and Use of Laboratory Animals, Directive 2010/63/EU of the European Parliament and of the Council of 22 September 2010 on the protection of animals used for scientific purposes, other relevant international standards and national legislation to protect animal welfare.

### 3.3. Preparation and Dosing of the Tested Compounds

The IRI solution used for the treatments was prepared by dissolving the drug in sterile 0.9% sodium chloride solution (Croatian Institute for Transfusion Medicine, Zagreb, Croatia). The tested dose of 100 mg/kg b.w. was selected adhering to the available literature [42,43,44,45].

THC was dissolved in sesame oil (Bio Primo, Ulm, Germany), and administered daily at a dose of 7 mg/kg b.w. [60,61,62].

### 3.4. Experimental Design

A total of 60 rats were randomly assigned to the following four groups. Group 1: control rats were given the same daily volume of the vehicle sesame oil as the THC-treated group. Group 2: rats administered IRI once, at day 0, via an intraperitoneal injection at a dose of 100 mg/kg b.w. Group 3: rats orally administered THC dissolved in sesame oil at a daily dose of 7 mg/kg b.w. for one, three and seven days, respectively. Group 4: rats administered IRI once, at day 0, via an intraperitoneal injection at a dose of 100 mg/kg b.w. and orally administered THC dissolved in the sesame oil at a daily dose of 7 mg/kg b.w. for one, three and seven days, respectively. Each group comprised 5 animals with minimal weight variation (300 g ± 10%). 

Rats had free access to food and drinking water. Their body weights were regularly monitored and the doses of THC were adjusted accordingly. Survival and clinical signs of intoxication were also evaluated on a daily basis. 

The experiment was terminated 24 h after the last treatment, when the rats were sacrificed using an anaesthetic cocktail (Narketan, Vetoquinol UK Ltd., Towcester, UK, 80 mg/kg b.w.; Xylapan, Vetoquinol UK Ltd., 12 mg/kg b.w., i.p.). Livers were removed, rinsed in cold saline, weighed, and processed for various analyses. 

Small pieces of freshly resected rat liver were put into chilled buffer [75 mM NaCl (Kemika, Zagreb, Croatia) and 24 mM Na_2_EDTA, pH 7.5] and minced with a pair of fine scissors [104] for a few minutes, into fine pieces, to release single cells. The obtained cellular suspension was immediately used for preparation of agarose microgels.

The blood samples were collected by dissection of carotid artery under general anaesthesia, allowed to clot for 2 h at room temperature, centrifuged (3000 rpm, i.e., 976× *g*, 2 × 15 min, at 4 °C), and serum for biochemical analyses was removed and stored at −20 °C. 

### 3.5. Body Weight, Liver Weight, and Calculation of Liver to Body Weight Ratio

Body weights and wet liver weights were recorded immediately after the experimental animals were sacrificed. Liver to body weight ratio was calculated using the formula: liver weight (g) × 100/body weight (g).

### 3.6. Biochemical Serum Analysis

Serum biochemical parameters included AST, ALT, bilirubin, ALP, and total protein. These were determined using a chemical analyser (Beckman Coulter AU 480; Brea, CA, USA). Activities of AST, ALT, and ALP were expressed as U/L. The amount of total protein was expressed as g/L. Bilirubin concentration (total bilirubin) was expressed as µmol/L.

### 3.7. Lipid Peroxidation

The concentration of thiobarbituric reactive substances (TBARS), as a measure of lipid peroxidation, was determined using a modification of the method by Drury et al. [120]. Butylated hydroxytoluene (BHT; 5 µL; 0.2% *w*/*v*) and phosphoric acid (750 µL; 1% *v*/*v*) were added to 50 µL samples. After mixing, 250 µL 0.6% (*w*/*w*) thiobarbituric acid (TBA) and 445 µL H_2_O were added and the reaction mixture was incubated in a water bath at 90 °C for 30 min. The mixture was cooled and absorbance was measured at 532 nm on a Shimadzu UV Probe Spectrophotometer (Kyoto, Japan). The concentration of TBARS was calculated using standard curves of increasing 1,1,3,3-tetramethoxypropane concentrations, and expressed as µmol/L.

### 3.8. Superoxide Dismutase Activity

Total superoxide dismutase (SOD) activity was measured spectrophotometrically according to Flohé and Ötting [121]. The reduction rate of cytochrome c by superoxide radicals was monitored at 550 nm utilizing the xanthine–xanthine oxidase system as the source for O_2_^•−^. SOD competed for the superoxide and decreased the reduction rate of cytochrome c. One unit of SOD was defined as the amount of enzyme that inhibits the rate of cytochrome c reduction by 50%. Enzyme activity was expressed as IU/g_protein_.

### 3.9. Catalase Activity

Catalase activity was measured at 25 °C, pH 7.0, at 240 nm [122]. Enzyme activity was calculated using the molar extinction coefficient (40.0 mM/cm), and expressed as IU/g_protein_.

### 3.10. Protein Quantification

Determination of protein concentration was carried out according to the method by Bradford [123] using bovine serum albumin as standard.

### 3.11. The Alkaline Comet Assay

The alkaline comet assay was performed according to standard procedure [124], with minor adjustments. To prepare agarose microgels, we used fully frosted precleaned microscope slides (Surgipath^®^, Cambridgeshire, UK) precoated with 0.6% normal melting point (NMP) agarose. Suspensions of liver cells (V = 10 µL per slide) were mixed with 0.5% low melting point (LMP) agarose, placed on slides and covered with a top layer of 0.5% LMP agarose. The microgels were lysed overnight at 4 °C in a freshly prepared buffer (2.5 mol/L NaCl (Kemika, Zagreb, Croatia), 100 mmol/L Na_2_EDTA, 10 mmol/L Tris-HCl, 1% Na-laurylsarcosinate, pH = 10) with 1% Triton X-100 and 10% dimethyl sulfoxide (Kemika, Zagreb, Croatia). After lysis, microgels were denatured (20 min) in alkaline denaturation/electrophoresis buffer (300 mM NaOH (Kemika, Zagreb, Croatia) and 1 mM Na_2_EDTA, pH > 13). Electrophoresis lasted for 20 min, at 4 °C, 25 V, and 300 mA. The microgels were then neutralised using a Tris-HCl buffer (0.4 mol/L; pH = 7.5). Before microscopic analysis, the slides were stained with ethidium bromide (20 µg/mL). They were analysed using an epifluorescence microscope (Olympus BX50, Tokyo, Japan) equipped with appropriate filters, at 200× magnification. The level of DNA damage in individual cells was estimated using Comet Assay IV™ software (Instem-Perceptive Instruments Ltd., Suffolk, Halstead, UK). One well-trained scorer performed all of the comet measurements on the coded/blinded slides. Random fields were selected and the comets were captured at a constant depth of the gel, avoiding edges and occasional dead cells. A total of 200 comets per rat were measured on replicate slides in two independent evaluations (altogether 1000 individual comet measurements per each experimental group were performed). Tail length (presented in micrometres) and tail intensity (i.e., DNA% in tail) were chosen as indicators of DNA damage.

### 3.12. Statistical Analysis

Statistical calculations were done using the Dell™ Statistica™ 13.2 software (StatSoft, Tulsa, OK, USA). The significance of the differences in body weight, liver weights, and serum markers of liver function between groups was tested using ANOVA and post hoc Tukey HSD test. The data acquired by alkaline comet assay were first evaluated using descriptive statistics. Further evaluations were performed by ANOVA. Before data analysis, to normalize data distribution log_10_-transformation was used to normalize the distribution and equalize variances. Finally, post hoc Tukey HSD test was used for calculations regarding pairwise comparisons. For biochemical markers of oxidative stress, Kruskal–Wallis test was used. The level of statistical significance was set at *p* < 0.05.

## 4. Conclusions

Although preliminary in nature, this study adds several potentially important findings to the existing knowledge on the subject. This is a pioneer study that quantified levels of primary DNA damage induced by THC in rat hepatocytes using the alkaline comet assay. No similar reports in that regard were available in the existing literature so far. In spite of the limitations set by our study design, the information given could be convincing to other researchers, for instance, to genetic toxicologists familiar with the use of alkaline comet assay.

The strength of our findings also lies in the fact that they provide evidence on synergistic enhancement of acute IRI toxicity by THC in liver of rats, at tested doses and applied treatment schedule, which was confirmed by body and liver weight reduction. Although single THC affected ALP and AP levels more than single IRI, after combined treatment, no significant impairments of liver function were noticed, which confirmed the high capacity of the liver to counteract the damage. Combined exposure also led to increased oxidative stress responses in 3-, and 7-day treatments compared to single IRI. While shorter exposures of rats to THC did not significantly impair DNA damage levels compared to control, continuous 7-day oral exposure caused minor DNA impairments in hepatocytes (significantly higher mean value of tail length compared to 1- and 3-day exposures). Concomitant intake of THC also slightly affected the levels of IRI genotoxicity, but not in a consistent manner.

Our results also point to different sensitivity of the biomarkers used in the assessments. At the tested doses and treatment schedule applied, the alkaline comet assay, which is based on the detection of primary DNA damage, was the most subtle biosensor that pointed to the adverse effects of mutual IRI and THC exposure in rat liver. Oxidative stress biomarkers also sensitively detected toxic effects of all of the tested compounds. However, the standard biochemical markers of liver function showed a somewhat lower sensitivity in the present study. Nevertheless, their role could be more vital in chronic exposures, as suggested by related studies.

In view of the methodological constraints of our research, which prevented us from confirming some of the assumptions derived from the obtained results, the findings of this study have to be proven in forthcoming studies using more advanced methods. Since biochemical methods provide a static assessment of the degree of liver damage, but do not give accurate information about residual liver function, to further elucidate changes in enzyme biomarkers following exposure to IRI and THC, future research, in this regard, should focus on more subtle molecular methods (for instance qPCR), to determine expression of particular genes of interest (especially for UGT1A1 and CYP3A4). An issue worth additional research at the cellular level is also the role of mitochondrial dysfunctions in hepatotoxicity, considering that newer studies imply IRI effects on mtDNA and impairments of mitochondrial DNA topoisomerases. Valuable information could also be obtained using histological and immunohistochemistry methods, as well as the methods for apoptosis detection, and we hope that our upcoming research will also extend in that direction.
